# Cross-presentation of the oncofoetal tumor antigen 5T4 from irradiated prostate cancer cells - a key role for Hsp70

**DOI:** 10.1186/2051-1426-2-S3-P161

**Published:** 2014-11-06

**Authors:** Josephine C Salimu, Lisa K Spary, Saly Al-Taei, Aled Clayton, Malcolm Mason, John Staffurth, Zsuzsanna Tabi

**Affiliations:** 1 Cardiff University, Cardiff, UK; 2 Velindre NHS Trust, Cardiff, UK

## 

Immune responses contribute to the success of radiation therapy of solid tumors; however, the mechanism of triggering CD8^+ ^T cell responses is poorly understood. Antigen cross-presentation from tumor cells by dendritic cells (DC) is a likely dominant mechanism to achieve CD8^+ ^T cell stimulation. We established a cross-presentation model in prostate cancer in which DC present a naturally expressed oncofoetal tumor antigen (5T4) from irradiated DU145 tumor cells to 5T4-specific T cells. Ionising radiation (12 Gy) caused G2/M cell cycle arrest and cell death, increased cellular 5T4 levels, induced passive release of high-mobility protein group-B1 (HMGB1) and upregulated surface calreticulin and Hsp70 expression in DU145 cells. Co-culture of DC with irradiated tumor cells lead to efficient phagocytosis of tumor cells and upregulation of CD86 and HLA-DR on DC. CD8^+ ^5T4-specific T cells, stimulated with these DC, proliferated and produced IFNγ. Inhibition of HMGB1 function decreased T cell stimulation but not DC activation, while TRIF/MyD88 inhibition only had a marginal effect on T cell stimulation. Unlike previous reports, we found no functional evidence that DC with Asp299Gly toll-like receptor-4 (TLR4) single nucleotide polymorphism had impaired ability to cross-present tumor antigen. However, we observed a highly significant and robust prevention of antigen cross-presentation when tumor cells were pre-treated with the novel Hsp70 inhibitor, VER155008. The inhibitor also prevented CD86 upregulation on DC co-cultured with irradiated tumor cells. Together, our study demonstrates that radiation induces immunologically relevant changes in tumor cells, which can trigger CD8^+ ^T cell responses via a predominantly Hsp70-dependent antigen cross-presentation process.

